# Altered Regional Cerebral Blood Flow and Brain Function Across the Alzheimer's Disease Spectrum: A Potential Biomarker

**DOI:** 10.3389/fnagi.2021.630382

**Published:** 2021-02-22

**Authors:** Qianqian Zhang, Qing Wang, Cancan He, Dandan Fan, Yao Zhu, Feifei Zang, Chang Tan, Shaoke Zhang, Hao Shu, Zhijun Zhang, Haixia Feng, Zan Wang, Chunming Xie

**Affiliations:** ^1^Department of Neurology, Affiliated ZhongDa Hospital, School of Medicine, Southeast University, Nanjing, China; ^2^Neuropsychiatric Institute, Affiliated ZhongDa Hospital, School of Medicine, Southeast University, Nanjing, China; ^3^The Key Laboratory of Developmental Genes and Human Disease, Southeast University, Nanjing, China; ^4^Department of Nursing, Affiliated ZhongDa Hospital, School of Medicine, Southeast University, Nanjing, China

**Keywords:** Alzheimer's disease, arterial spin labeling, resting-state functional MRI, regional homogeneity, cerebral blood flow, amplitude of low frequency fluctuation

## Abstract

**Objective:** To investigate variation in the characteristics of regional cerebral blood flow (rCBF), brain activity, and intrinsic functional connectivity (FC) across the Alzheimer's disease spectrum (ADS).

**Methods:** The study recruited 20 individuals in each of the following categories: Alzheimer's disease (AD), mild cognitive impairment (MCI), subjective cognitive decline (SCD), and healthy control (HC). All participants completed the 3.0T resting-state functional MRI (rs-fMRI) and arterial spin labeling scans in addition to neuropsychological tests. Additionally, the normalized CBF, regional homogeneity (ReHo), and amplitude of low-frequency fluctuation (ALFF) of individual subjects were compared in the ADS. Moreover, the changes in intrinsic FC were investigated across the ADS using the abnormal rCBF regions as seeds and behavioral correlations. Finally, a support-vector classifier model of machine learning was used to distinguish individuals with ADS from HC.

**Results:** Compared to the HC subjects, patients with AD showed the poorest level of rCBF in the left precuneus (LPCUN) and right middle frontal gyrus (RMFG) among all participants. In addition, there was a significant decrease in the ALFF in the bilateral posterior cingulate cortex (PCC) and ReHo in the right PCC. Moreover, RMFG- and LPCUN-based FC analysis revealed that the altered FCs were primarily located in the posterior brain regions. Finally, a combination of altered rCBF, ALFF, and ReHo in posterior cingulate cortex/precuneus (PCC/PCUN) showed a better ability to differentiate ADS from HC, AD from SCD and MCI, but not MCI from SCD.

**Conclusions:** The study demonstrated the significance of an altered rCBF and brain activity in the early stages of ADS. These findings, therefore, present a potential diagnostic neuroimaging-based biomarker in ADS. Additionally, the study provides a better understanding of the pathophysiology of AD.

## Introduction

Alzheimer's disease (AD) is one of the most important public health burdens worldwide. Notably, constant frustrations in drug development against the disease revealed the complexity of the pathogenic mechanism of AD. This, therefore, suggested that a more comprehensive study of specific neurobiological changes should be performed across the Alzheimer's disease spectrum (ADS), at both the preclinical and clinical stages. Moreover, the search for effective biomarkers is essential for the implementation of effective interventions before the development of significant neuronal damage.

Multimodal MRI has extensively been applied to investigate the abnormalities in brain structure and function in the ADS. In addition, the estimation of medial temporal lobe atrophy (MTA) by structural MRI (sMRI) is used as a neuroimaging biomarker in the diagnosis of AD (Ten et al., 2017). However, the obvious brain structure atrophy might imply the appearance of irreversible neuronal damage in the process of AD pathophysiology. In contrast, the resting-state functional MRI (rs-fMRI) can detect early functional changes in the brain reflected by the intrinsic blood-oxygen-level-dependent (BOLD) signals before the appearance of cognitive decline and brain structural atrophy (Galvin et al., 2011; Habib et al., 2017; Kawagoe et al., 2019). Moreover, several brain regions, including the hippocampus (HIP), posterior cingulate cortex (PCC), precuneus (PCUN), prefrontal cortex (PFC), temporal lobe, and angular gyrus (ANG) (Kawagoe et al., 2019; Xue et al., 2019; Zheng et al., 2019), have been reported as the core hub of brain networks involved in the pathophysiology of AD. Importantly, functional changes in the brain are independent of and even more sensitive than brain structure atrophy during the early stages of AD (Galvin et al., 2011; Xie et al., 2015; Kawagoe et al., 2019).

In addition, arterial spin labeling (ASL), utilizing intravascular water as an endogenous contrast agent, can measure regional cerebral blood flow (rCBF) (Ma et al., 2017). Notably, several studies consistently reported that rCBF displayed a decreasing trend with the progression of cognitive impairment in ADS (Binnewijzend et al., 2013; Ding et al., 2014; Trebeschi et al., 2016; Ma et al., 2017; Li et al., 2020). Moreover, compared to the age-matched subjects of health control (HC) and subjects of subjective cognitive decline (SCD), subjects of the mild cognitive impairment (MCI) and patients with AD presented decreased perfusion in the parietal lobe, PCC/PCUN, and occipital lobe (Binnewijzend et al., 2013; Ding et al., 2014; Trebeschi et al., 2016; Ma et al., 2017; Duan et al., 2020). Furthermore, the perfusion patterns identified by ASL were highly congruent with that provided by PET (Schroeter et al., 2009; Riederer et al., 2018; Dolui et al., 2020). PCC/PCUN, as one of the core regions in the default mode network (DMN), has been widely reported to be associated with a decreased rCBF, disrupted activity, and a destructive brain network in subjects with MCI and subjects with AD (Yoshiura et al., 2009; Sierra-Marcos, 2017; Xue et al., 2019). Notably, functional abnormalities of PCC/PCUN were also associated with the increased amyloid burden and decreased hippocampal volume (Khan et al., 2020). Additionally, a recent study, which explored the correlation between an altered rCBF and brain function in AD, revealed that the combination of ASL and the amplitude of low-frequency fluctuations (ALFF) in the PCC/PCUN could be used as a potential biomarker for the diagnosis of AD (Zheng et al., 2019). However, whether the integration of an altered rCBF and functional parameters in PCC/PCUN is capable of predicting various stages of AD is still unclear.

Therefore, the present study aimed to investigate the altered brain perfusion and function in all phases of ADS. First, the study measured whole-brain rCBF, regional homogeneity (ReHo), and the ALFF in each subject through the ASL and rs-fMRI approaches. Second, a partial correlation analysis was performed between the altered regions of each modality image and neuropsychological tests in ADS to obtain the behavioral significance of these altered brain functions. Third, the study investigated the changes in whole-brain functional connectivity of the identified rCBF regions as a seed in the ADS. Finally, the altered rCBF, ALFF, and ReHo in PCC/PCUN were integrated to get an imaging biomarker for the prediction of the ADS using a linear support vector machine (SVM) based on the machine learning approach.

## Materials and Methods

### Participants

Participants were recruited from media advertisements and neurology outpatient clinics of the Affiliated Zhongda Hospital, Southeast University (Nanjing, China). All the subjects and their relatives were then provided with all relevant details before signing a written informed consent to participate in the study. A total of 80 Han Chinese individuals from eastern China were included in the study. Additionally, the participants underwent a full neuropsychological test battery, physical examination, blood tests, and a multi-modal MRI brain scan. This study was approved by the Research Ethics Committee of the Affiliated Zhongda Hospital, Southeast University (Nanjing, China).

### Neuropsychological Assessments

Comprehensive cognitive function assessment and neurological examination were conducted on all the participants by two experienced neuropsychiatrists. The items specifically included activities of daily living (ADL), the Hamilton Depression Scale (HAMD), the Hachinski Ischemic Scale (HIS), the Mini-Mental State Examination (MMSE), and additional tests that covered the four previously characterized cognitive domains, namely: memory (episodic memory), information processing speed, visuospatial function, and executive function. In addition, memory tests included the Auditory Verbal Learning Test-20-min-Delayed Recall (AVLT-20-min-DR), Logical Memory Test-20-min-Delayed Recall (LMT-20-min-DR), and the Rey–Osterrieth Complex Figure Test-20-min-Delayed Recall (ROCFT-20-min-DR). On the other hand, the information processing speed was measured using the Symbol Digit Modalities Test (DSST), Trail Making Tests-A (TMT-A), and the Stroop Color and Word Test A and B. Moreover, the visuospatial function domain included the Clock Drawing Test (CDT) and the ROCFT. Additionally, the executive function domain was measured using the Stroop Color and Word Test C, the Digit Span Test (DST), the Verbal Fluency Test (VFT), the Trail Making Tests-B (TMT-B), and the Semantic Similarity Test (Shi et al., 2019). Finally, the raw scores of each test were transformed into *z*-scores using the mean and SD in order to calculate the composite score of each cognitive domain (Xie et al., 2012).

### Inclusion and Exclusion Criteria

All subjects were independently evaluated and diagnosed by two experienced neuropsychiatrists. Participants were required to meet the following criteria: (1) 55–85 years old, (2) educational years ≥8, (3) right-handed, and (4) should have been from the Han Chinese population. In addition, the inclusion criteria for HC contained: (1) no memory complaints and normal in ADL, (2) all neuropsychological tests were within the normal range, and (3) no abnormal findings in routine brain MRI (Dubois et al., 2014; Yan et al., 2018). On the other hand, the eligibility criteria for SCD contained: (1) frequent complaints of memory problems; (2) normal neuropsychological performance of age- and education-matched norms; and (3) lack of impairments in the ADL (Dubois et al., 2014; Yan et al., 2018). Moreover, the inclusion criteria for MCI contained: (1) complaints of memory impairment for more than 3 months; (2) MMSE score ≥24 and HAMD ≤ 7; (3) objective impairment in at least one cognitive domain, AVLT-20-min-DR score within ≤ 1.5 SD of the same age- and education-adjusted norms (cut-off of ≤ 4 correct responses on 12 items for subjects); and (4) no dementia (Dunn et al., 2014; Shi et al., 2019). The clinical diagnosis of AD was based on the criteria by the National Institute of Neurological and Communicative Disorders and Stroke and the Alzheimer's Disease and Related Disorders Association (NINCDS-ADRDA). They included (1) a clear-cut history of worsening cognition over 6 months; (2) MMSE score <24; (3) impairments in the ADL; and (4) dementia (McKhann et al., 2011; Arevalo-Rodriguez et al., 2015).

On the other hand, the exclusion criteria were as follows: (1) history of serious neurological and psychiatric diseases including major depressive disorders, schizophrenia, hydrocephalus, significant cerebrovascular disorders, and brain trauma; (2) systemic illnesses, such as uncontrolled hypertension, diabetes, abnormalities in the thyroid hormone, folic acid levels, vitamin B12, or significant liver and kidney diseases; and (3) the inability to undergo an MRI scan (Xie et al., 2012; Shi et al., 2019).

### Acquisition of MRI Data

All MRI data was obtained using the Siemens Verio 3Tesla MRI Scanner with an 8-channel head-coil. In addition, the rs-fMRI images were obtained using the following parameters: 240 time points, repetition time (TR) = 2,000 ms, echo time (TE) = 25 ms, flip angle = 90°, number of slices = 36, slice thickness = 4 mm, spatial resolution = 3.75 × 3.75 × 4 mm^3^, acquisition matrix = 64 × 64, and field of view (FOV) = 240 × 240 mm^2^. Additionally, the 3D magnetization-prepared rapid gradient echo (MP-RAGE) were acquired to get the T1-weighted images with the following data parameters: TR = 1,900 ms, TE = 2.48 ms, slice thickness = 1 mm, FA = 90°, FOV = 256 × 256 mm, gap = 0 mm, and number of slices = 176. Moreover, the ASL data was obtained using the following parameters: TI1 = 600 ms, TI2 = 1.6 s, flip angle = 90°, number of slices = 27, slice thickness = 4.0 mm, TR = 4 s, TE = 12 ms, FOV = 220 × 220 mm^2^, and matrix size = 64 × 64.

### Data Pre-processing

SPM8 software (http://www.fil.ion.ucl.ac.uk/spm) was used to analyze the T1-weighted and ASL images. First, the T1-weighted images were segmented into three parts [cerebrospinal fluid (CSF), gray matter (GM), and white matter] using the VBM8 toolbox (http://dbm.neuro.uni-jena.de/wordpress/vbm/). Out of these, the segmented GM volume was normalized and regressed out as a covariate to control the effects of GM volume on the analysis of rCBF, ALFF, ReHo, and FC. Thereafter, deformation matrices were used to co-register the ASL images to the corresponding native GM images, which were spatially normalized to the Montreal Neurological Institute (MNI) space. During spatial normalization, the ASL images were resampled into 2 × 2 × 2 mm^3^ voxel size. Finally, the resulting ASL images were smoothened using an isotropic 6 mm Gaussian filter for subsequent multiple comparison analysis.

Additionally, Data Processing & Analysis for Brain Imaging (DPABI, http://www.rfmri.org) was used to perform ALFF, ReHo, and seed-based FC analysis on the rs-fMRI images (Yan et al., 2016). Briefly, the first 10 volumes for each subject were removed in case of possible instability in the rs-fMRI signal. The remaining 230 points in time were then corrected for timing differences before adopting the Friston 24-parameter model to regress out the effects of head motion from realignment (Qi et al., 2020). All subjects with cumulative translation and rotation of head motion were <2 mm or 2°. Thereafter, the original space was registered to the MNI space with a resampled voxel size of 3 mm isotropic by using the DARTEL templates created during the preprocessing of T1-weighted images (Ashburner, 2007), which could alleviate the interference from different brain structures between subjects. Following this, the normalized images were smoothed with a 6 × 6 × 6 mm Gaussian kernel to reduce variation. The effects of confounding factors were then removed and they included the global mean signal, CSF signal, and white matter signal (Zheng et al., 2019). Finally, the previously generated images were filtered between 0.01 and 0.08 Hz so as to control noise interferences.

Moreover, a seed-based connectivity analysis was adopted to investigate FC changes in the ADS. Regions showing significant differences in rCBF among the groups were selected as regions of interest (ROIs). The mean time series of the seed regions was extracted for each participant and correlated with each voxel of the whole brain to obtain the seed-based FC maps, which were then transformed to *z*-maps based on the Fisher *z*-transformation (Waltz et al., 2013; Wang et al., 2019).

### Statistical Analyses

The analysis of variance (ANOVA) and chi-square tests were used for demographic characteristics. Additionally, the mixed analysis of covariance (ANCOVA) was used to calculate differences in clinical scores and images among subjects (*p* < 0.05, SPSS 20.0) after controlling for age, gender, and education. For image data analysis (voxel-wised ANCOVA), GM volume, age, and education were controlled as covariates of no interest, and the Gaussian random field (GRF) theory was used for multiple comparison correction (cluster level *p* < 0.05, voxel-level *p* < 0.001). *Post hoc* analysis was also performed with the Bonferroni correction to evaluate differences between the four groups (*p* < 0.05).

Thereafter, a partial correlation analysis was used to investigate relationships between the behavioral scores and the altered rCBF, functional activity, and connectivity in all the subjects, after controlling for age, gender, and education as nuisance covariates (*p* < 0.05/5 = 0.01).

Finally, an SVM model based on the machine learning approach was adopted to obtain an imaging biomarker for the classification of the ADS by integrating the altered rCBF, ALFF, and ReHo in specific regions. Briefly, we used an SVM package, which was built in MATLAB, the LIBSVM toolbox to get optimal classifiers and test the power of classification (Pirooznia and Deng, 2006). The mean values of PCC/PCUN that showed significant group differences in rCBF, ALFF, and ReHo were employed as input features, which were all based on voxel-wise measures. Due to our limited sample size, the leave-one-out cross-validation (LOOCV) was used to quantify the power of classification (Wee et al., 2011). At last, receiver operating characteristic (ROC) curves were utilized to assess the performance of the classifier using the results from the LOOCV data. The classification performance was manifested at the area under the ROC curve (AUC), and the larger the AUC, the better the performance. Detailed information can be found in the Supplementary Material.

## Results

### Demographic and Neuropsychological Tests

Table 1, Supplementary Table 1, and Supplementary Figure 1 show the main demographic and clinical scores of all the subjects. There were no significant differences in age, gender, and education, as well as HAMD and HIS scores (*p* > 0.05), among the subjects. However, there were obvious differences in the MMSE, ADL, and composite *z*-scores of each cognitive domain between AD and the other three groups. *Post hoc* analysis revealed that the AD groups showed the worst behavioral performance when compared with the other three groups. In addition, more importantly, the four groups showed significant differences with each other in the episodic memory scores which decreased with disease severity (i.e., HC > SCD > MCI > AD).

**Table 1:** Comparison of demographic, clinical characteristics, and cognitive function in all subjects.

	**HC (*n* = 20)**	**SCD (*n* = 20)**	**MCI (*n* = 20)**	**AD (*n* = 20)**	***p-*Value**
Age (years)	70.75 ± 5.37	68.60 ± 7.30	71.95 ± 5.93	73.00 ± 6.03	0.144
Gender (F/M)	8/12	16/4	12/8	10/10	0.067^†^
Education (years)	13.38 ± 2.89	12.35 ± 3.63	11.40 ± 3.73	10.75 ± 3.52	0.096
HAMD scores	0.50 ± 0.89	2.40 ± 3.96	2.85 ± 2.96	2.55 ± 3.46	0.176
HIS scores	1.75 ± 0.85	1.05 ± 1.32	1.60 ± 1.35	1.70 ± 1.26	0.459
ADL scores	20.00 ± 0.00	20.10 ± 0.45	20.15 ± 0.37	26.45 ± 7.20^cef^	<0.001
MMSE scores	28.60 ± 1.19	28.80 ± 1.28	26.95 ± 2.09	19.60 ± 3.27^cef^	<0.001
**Composite** ***z*****-scores of each cognitive domain**					
Episodic memory	2.75 ± 1.27	1.37 ± 1.86^a^	−1.51 ± 1.16^bd^	−2.85 ± 0.94^cef^	<0.001
Visuospatial function	0.64 ± 0.66	0.50 ± 1.16	0.13 ± 1.20	−1.19 ± 2.57^ef^	0.002
Information processing speed	2.46 ± 2.31	1.16 ± 2.23	0.32 ± 2.83	−4.10 ± 2.71^cef^	<0.001
Executive function	2.49 ± 1.79	0.84 ± 1.98	0.62 ± 1.92^b^	−4.35 ± 2.61^cef^	<0.001

The values of p were obtained from the one-way ANOVA (age and education) and the ANCOVA (controlled for age, gender, and education) analysis, while the

† *p-value was obtained from the χ^2^ test. Data are presented as mean ± SD. Significant differences were found in MMSE, ADL, and four cognitive domains among all the groups. Post hoc analysis (Bonferroni correction) further revealed the source of ANCOVA differences: (a) HC vs. SCD; (b) HC vs. MCI; (c) HC vs. AD; (d) SCD vs. MCI; (e) SCD vs. AD; (f) MCI vs. AD. HC, Healthy Control; SCD, Subjective Cognitive Decline; MCI, Mild Cognitive Impairment; AD, Alzheimer's Disease; F/M, Female/Male; MMSE, Mini-Mental State Examination; ADL, Activities of Daily Living; HAMD, Hamilton Depression Scale; HIS, Hachinski Ischemic Scale*.

### Differences in Brain rCBF in the Four Groups

**Figure 1:**
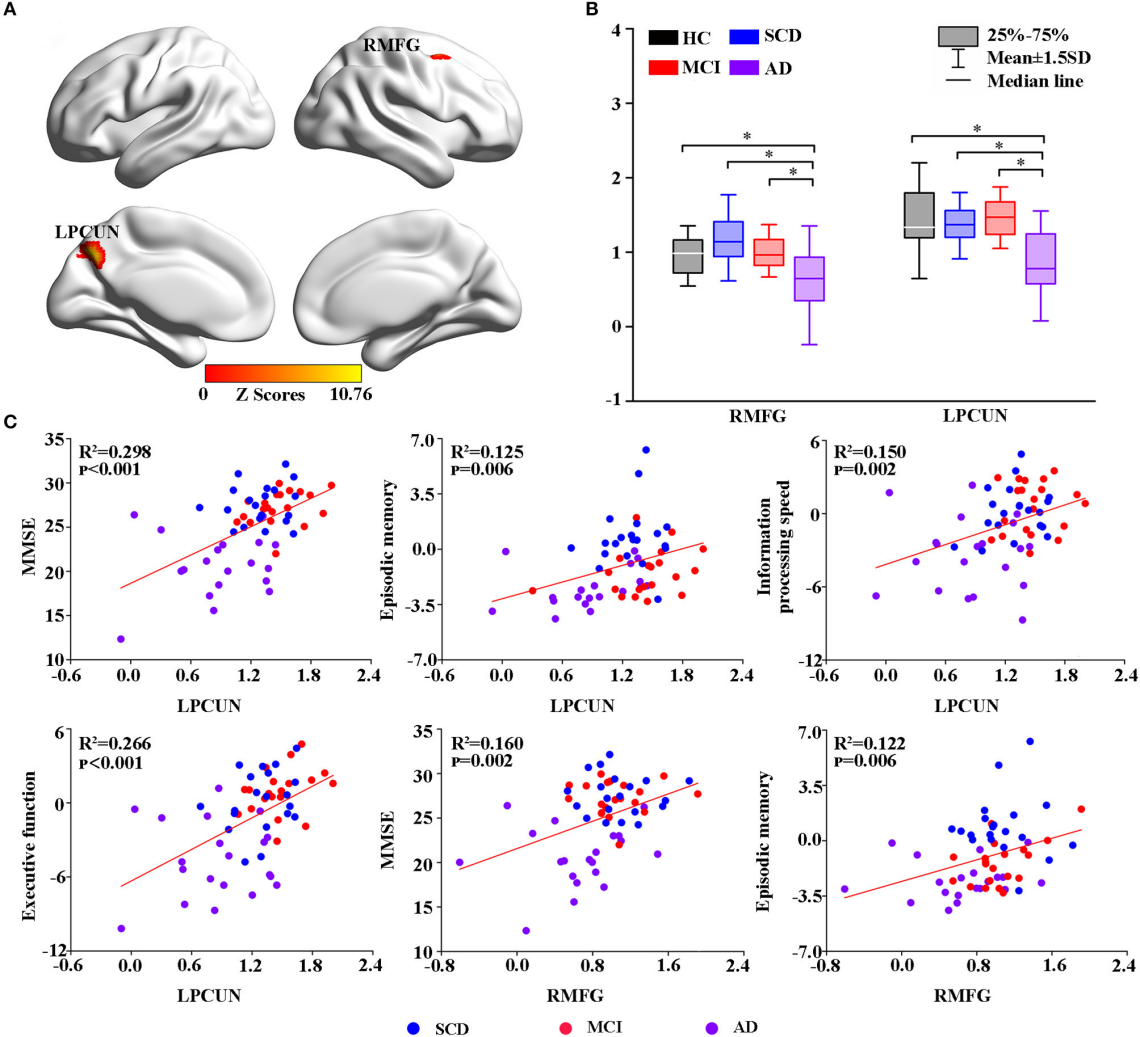
Group-level differences in rCBF among all subjects and behavioral significance. **(A)** The result revealed altered rCBF regions among the groups including LPCUN and RMFG (GRF-corrected, cluster level *p* < 0.05, voxel-level *p* < 0.001). The color bar represents the *z*-scores. **(B)** *Post hoc* analysis of the altered rCBF in the LPCUN and RMFG regions in all the groups (Bonferroni correction, *p* < 0.05). Box plots show the 25 percentile, the median, and the 75 percentile, and whisker plots are in mean ± SD. *Represents statistical difference (*p* < 0.05). **(C)** Significant correlations between the altered rCBF and cognitive performance in the SCD, MCI, and AD groups after controlling the effects of age, gender, education, and GM volumes as covariates of no interest (*p* < 0.01). AD, Alzheimer's Disease; MCI, Mild Cognitive Impairment; SCD, Subjective Cognitive Decline; HC, Healthy Control; MMSE, Mini-Mental State Examination; rCBF, Regional Cerebral Blood Flow; LPCUN, Left Precuneus; RMFG, Right Middle Frontal Gyrus; GM, Gray Matter; GRF, Gaussian Random Field.

The voxel-wise ASL analysis showed that the altered rCBF was primarily located in the left PCUN (LPCUN) and the right middle frontal gyrus (MFG) in the four groups (Figure 1A). In addition, *post hoc* analysis indicated that patients with AD suffered the most severe CBF loss in both altered brain regions compared to the other three groups (Figure 1B). Figure 1C shows the correlations between the different brain rCBF regions and clinical tests in the SCD, MCI, and AD groups. The rCBF in the LPCUN showed a clear positive association with MMSE (R^2^ = 0.298, *p* < 0.001), episodic memory (R^2^ = 0.125, *p* = 0.006), information processing speed (R^2^ = 0.150, *p* = 0.002), and executive function (R^2^ = 0.266, *p* < 0.001). However, it was only related to MMSE (R^2^ = 0.160, *p* = 0.002) and episodic memory (R^2^ = 0.122, *p* = 0.006) (adjusted *p*-values were < 0.01) in the right MFG. Notably, the higher the neuropsychological scores, the higher the rCBF value was in these regions of the brain.

### Altered Brain Activities Among Groups

**Figure 2:**
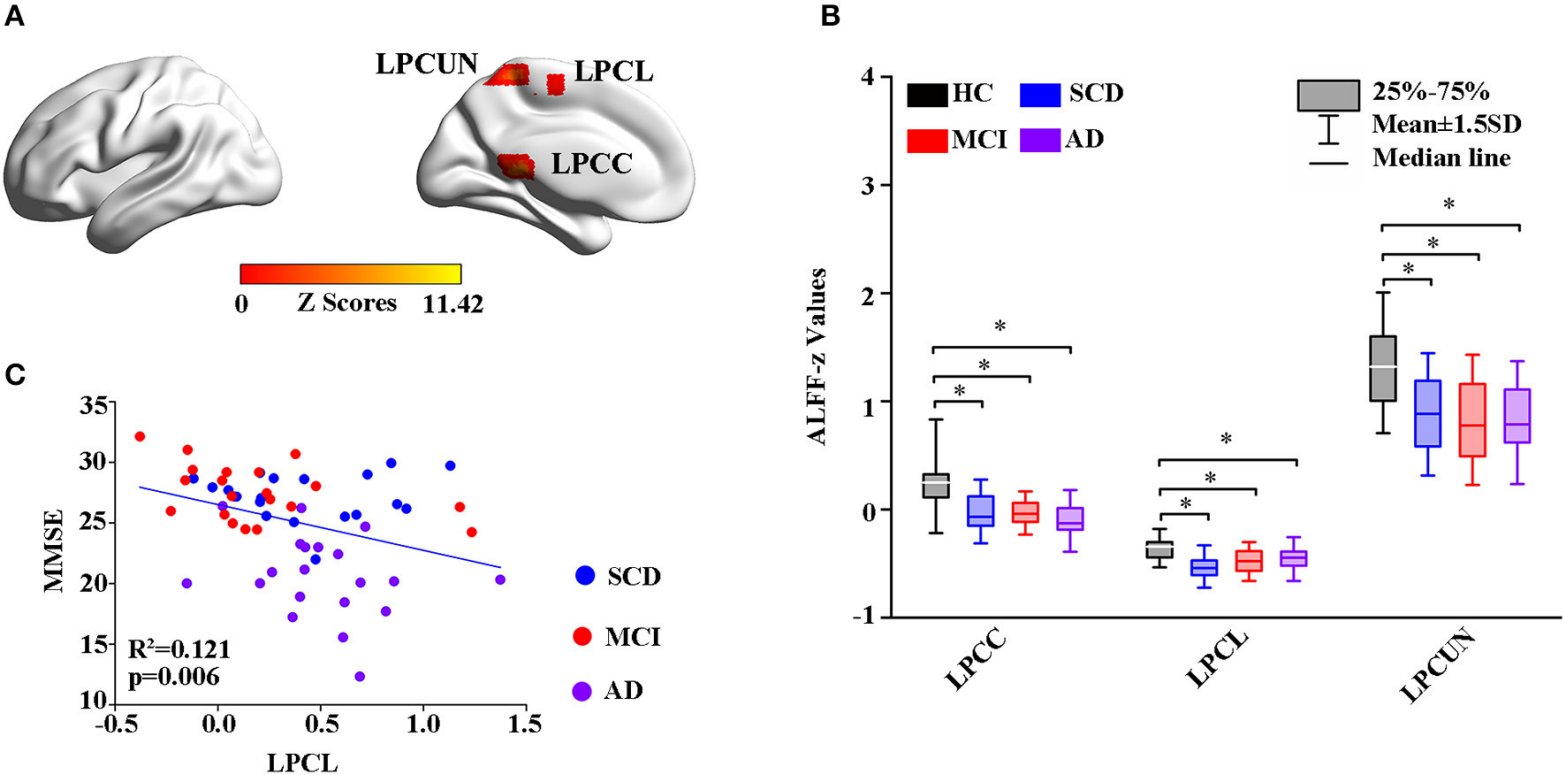
Group-level differences in ALFF among the subjects and behavioral significance. **(A)** The results showed the altered ALFF regions among the groups including LPCUN, LPCL, and LPCG (GRF-corrected, cluster level *p* < 0.05, voxel-level *p* < 0.001). Color bars represent the *z*-scores. **(B)** *Post hoc* analysis for the altered ALFF regions in all the groups (Bonferroni correction, *p* < 0.05). Box plots show the 25 percentile, the median, and the 75 percentile, and whisker plots are in mean ± SD. *Represents statistical difference (*p* < 0.05). **(C)** Significant correlations between the altered ALFF and neuropsychological tests in the SCD, MCI, and AD groups after controlling the effects of age, gender, education, and GM volumes as covariates of no interest (*p* < 0.01). AD, Alzheimer's Disease; MCI, Mild Cognitive Impairment; SCD, Subjective Cognitive Decline; HC, Healthy Control; MMSE, Mini-Mental State Examination; ALFF, Amplitude of Low-Frequency Fluctuation; LPCUN, Left Precuneus; LPCL, Left Paracentral Lobule; BPCG, Bilateral Posterior Cingulate Gyrus; GM, Gray Matter; GRF, Gaussian Random Field.

**Figure 3:**
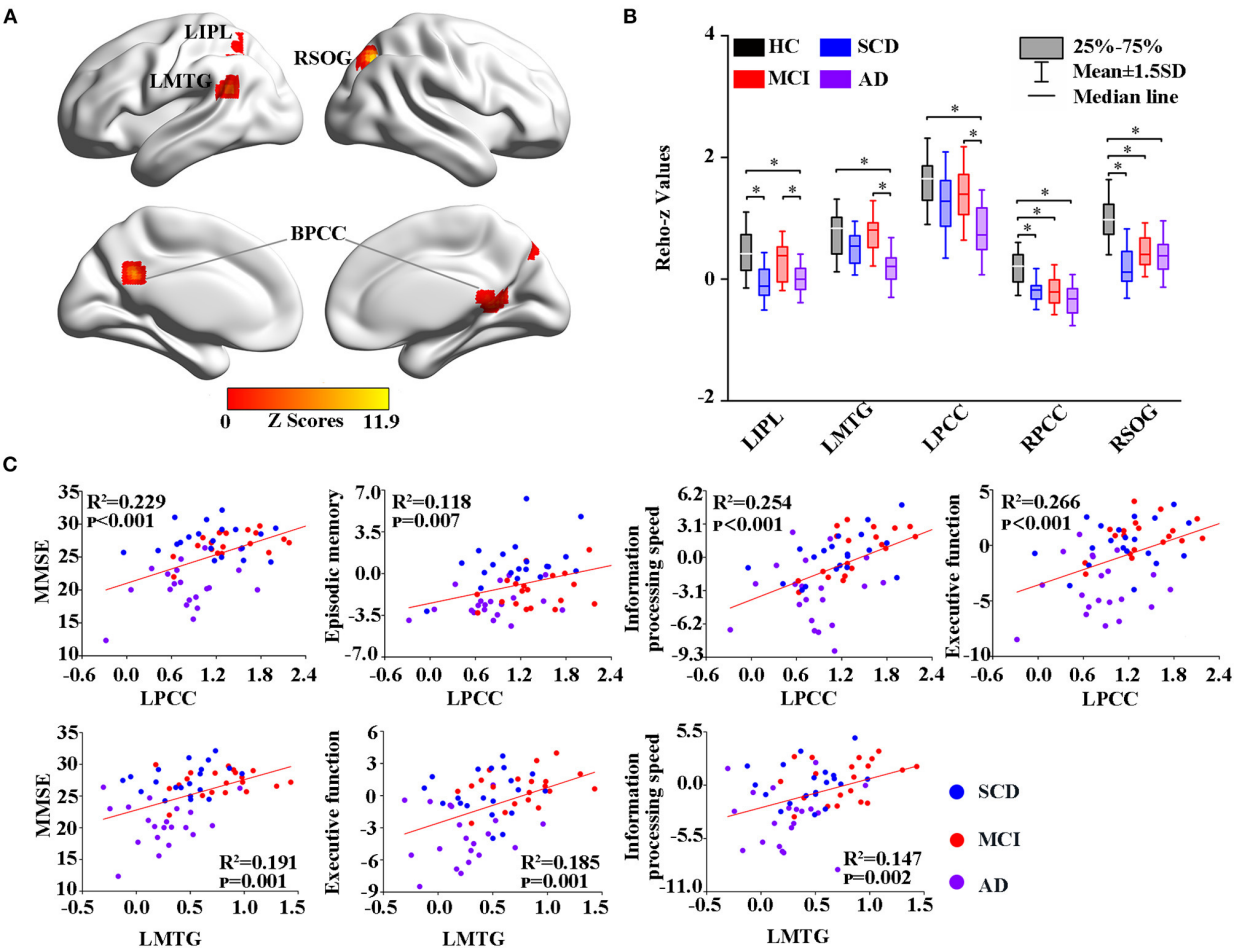
Group-level differences in ReHo among all the subjects and behavioral significance. **(A)** The results showed the altered ReHo regions among the groups, including BPCG, LIPL, LMTG, and RSOG (GRF-corrected, cluster level *p* < 0.05, voxel-level *p* < 0.001). **(B)** *Post hoc* analysis of the altered ReHo regions in all the groups (Bonferroni correction, *p* < 0.05). Box plots show the 25 percentile, the median, and the 75 percentile, and whisker plots are in mean ± SD. *Represents statistical difference (*p* < 0.05). **(C)** Significant correlations between the altered ReHo and neuropsychological tests in the SCD, MCI, and AD groups after controlling the effects of age, gender, education, and GM volumes as covariates of no interest (*p* < 0.01). AD, Alzheimer's Disease; MCI, Mild Cognitive Impairment; SCD, Subjective Cognitive Decline; HC, Healthy Control; MMSE, Mini-Mental State Examination; ReHo, Regional Homogeneity; BPCG, Bilateral Posterior Cingulate Gyrus; LIPL, Left Inferior Parietal; LMTG, Left middle Temporal gyrus; RSOG, Right Superior Occipital Gyrus; GM, Gray Matter; GRF, Gaussian Random Field.

Thereafter, the study measured intrinsic brain activity in each region using ALFF and ReHo (Figures 2, 3). Significant changes in ALFF were shown in the bilateral PCC, LPCUN, and left paracentral lobule (PCL) as shown in Figure 2A. Additionally, ReHo was significantly altered in the bilateral PCUN, left inferior parietal (IPL), left middle temporal gyrus (MTG), and right superior occipital gyrus (SOG) among the groups (Figure 3A). Moreover, the groups with the disease showed decreased ALFF and ReHo compared to the HC group (Figures 2B, 3B). It is also worth noting that ReHo significantly increased in the MCI group in most of the altered brain regions except for the right PCUN (Figure 3B). Furthermore, partial correlation analysis revealed the relationship between brain activity and the neuropsychological scores (adjusted *p*-values were < 0.01) as shown in Figures 2C, 3C. The results revealed a negative correlation between the ALFF in the LPCL and MMSE (R^2^ = 0.121, *p* = 0.006), as indicated in Figure 2C. In contrast, ReHo was positively associated with MMSE (LPCUN: R^2^ = 0.229, *p* < 0.001; LMTG: R^2^ = 0.191, *p* = 0.001), information processing speed (LPCUN: R^2^ = 0.254, *p* < 0.001; LMTG: R^2^ = 0.147, *p* = 0.002), and executive function (LPCUN: R^2^ = 0.266, *p* < 0.001; LMTG: R^2^ = 0.185, *p* = 0.001) in the LPCUN and left MTG as shown in Figure 3C. Moreover, there was a significant positive correlation between the ReHo of the LPCUN and the episodic memory (R^2^ = 0.118, *p* = 0.007), as highlighted in Figure 3C.

### Functional Connectivity Changes in the Resting State

**Figure 4:**
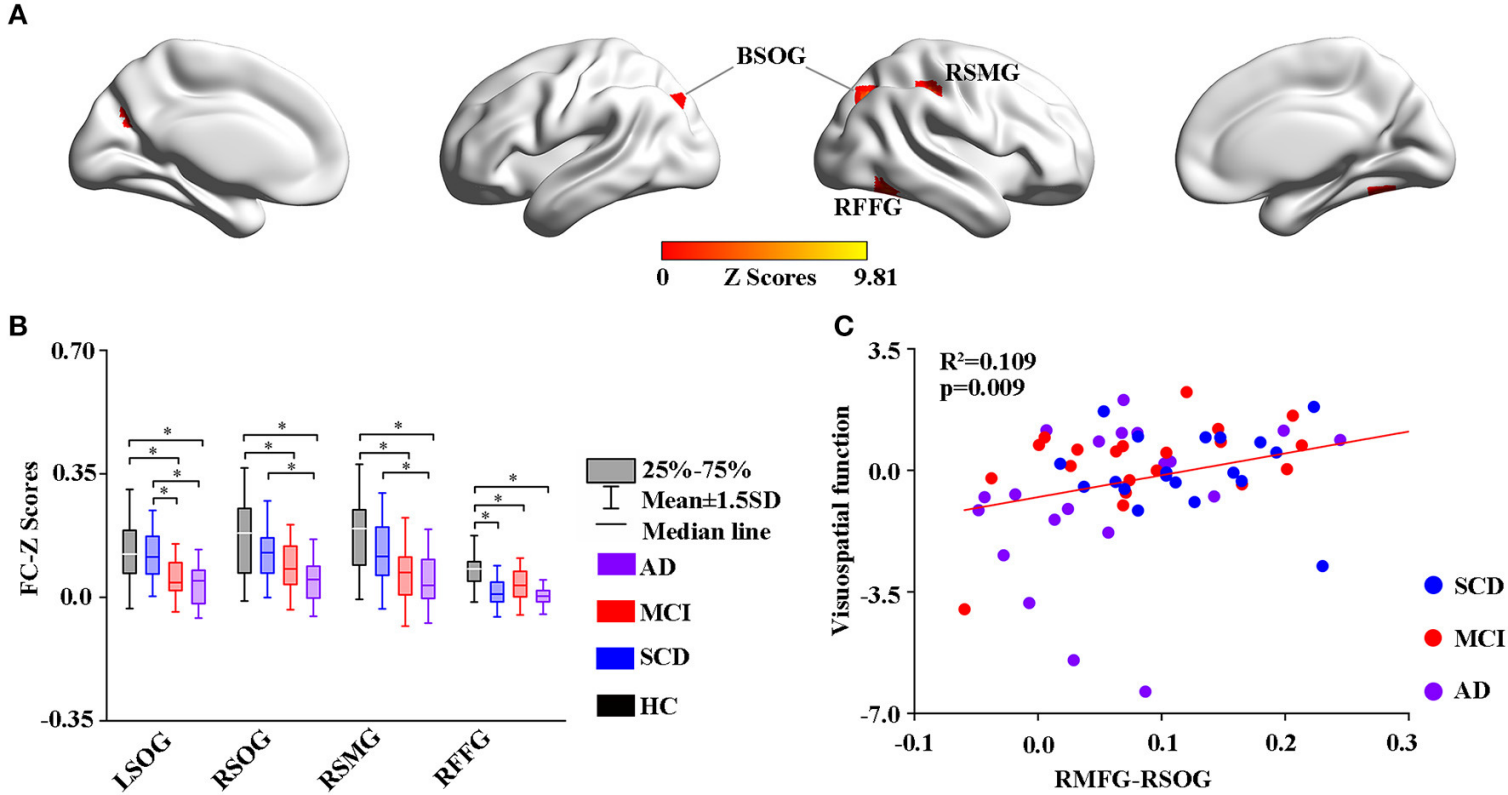
Group-level differences in RMFG-based functional connectivity among the subjects and behavioral significance. **(A)** The results showed the altered FC brain regions among the groups including BSOG, RITG, and RSMG (GRF-corrected, cluster level *p* < 0.05, voxel-level *p* < 0.001). **(B)** *Post hoc* analysis of the altered FC regions in all the groups (Bonferroni correction, *p* < 0.05). Box plots show the 25 percentile, the median, and the 75 percentile, whisker plots are in mean ± SD. *Indicates statistical difference (*p* < 0.05). **(C)** Significant correlations between the altered FC and cognitive performance in the SCD, MCI, and AD groups (*p* < 0.01) after controlling the effects of age, gender, education, and GM volumes as covariates of no interest (*p* < 0.01). AD, Alzheimer's Disease; MCI, Mild Cognitive Impairment; SCD, Subjective Cognitive Decline; HC, Healthy Control; FC, Function Connectivity; BSOG, Bilateral Superior Occipital Gyrus; RFFG, Right Fusiform Gyrus; RSMG, Right Supramarginal Gyrus; GM, Gray Matter; GRF, Gaussian Random Field.

**Figure 5:**
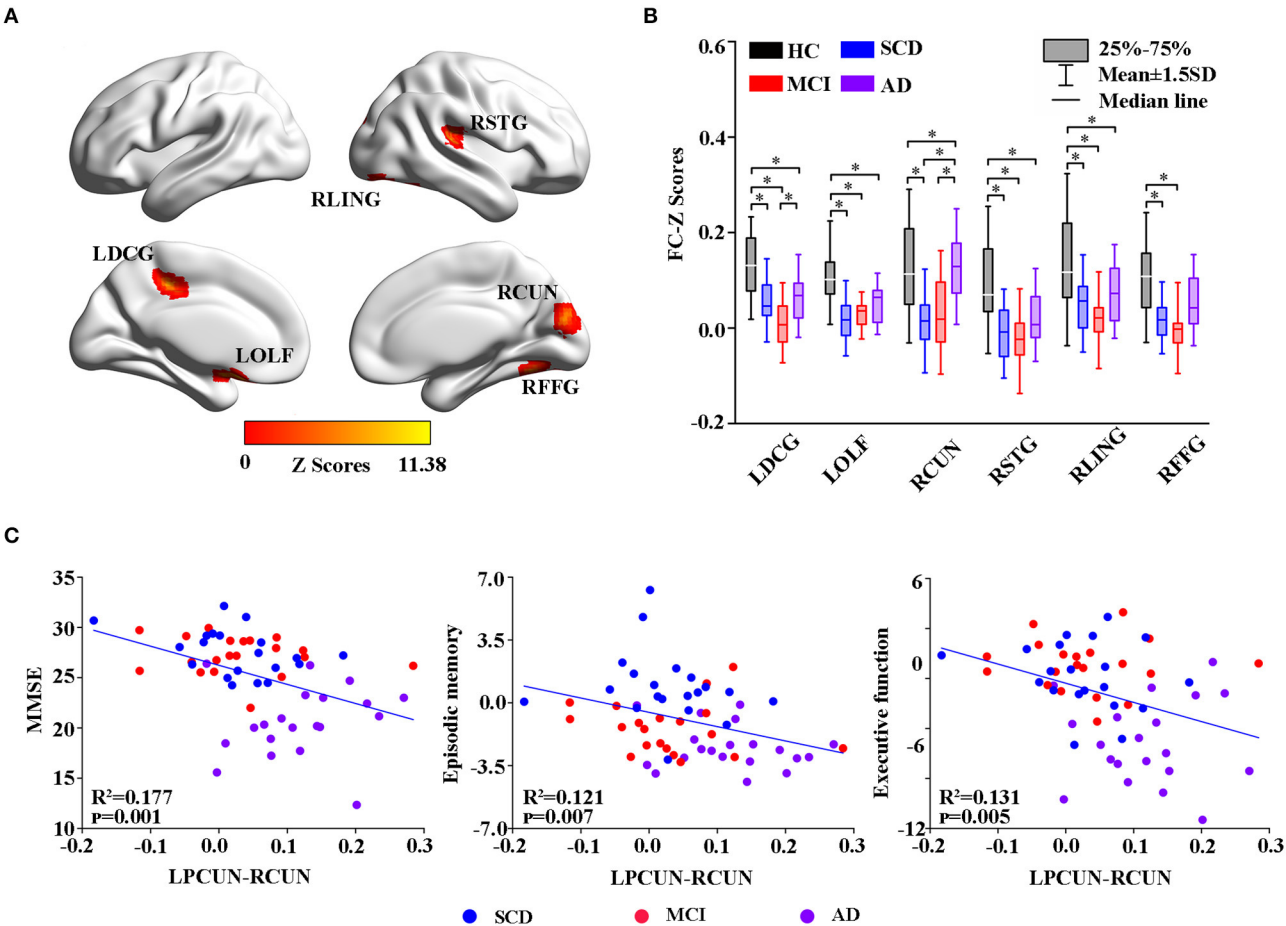
Group-level differences in the LPCUN-based functional connectivity across the subjects and behavioral significance. **(A)** The results showed the altered FC brain regions among the groups including LDCG, LOLF, RCUN, RSTG, RLING, and RFFG (GRF-corrected, cluster level *p* < 0.05, voxel-level *p* < 0.001). **(B)** *Post hoc* analysis of the altered FC regions in all the groups (Bonferroni correction, *p* < 0.05). Box plots show the 25 percentile, the median, and the 75 percentile, whisker plots are in mean ± SD. *Indicates statistical difference (*p* < 0.05). **(C)** Significant correlations between the altered FC and neuropsychological tests in the SCD, MCI, and AD groups after controlling the effects of age, gender, education, and GM volumes as covariates of no interest (*p* < 0.01). AD, Alzheimer's Disease; MCI, Mild Cognitive Impairment; SCD, Subjective Cognitive Decline; HC, Healthy Control; FC, Function Connectivity; LDCG, Left Median Cingulate and Paracingulate Gyri; LOLF, Left Olfactory Cortex; RCUN, Right Cuneus; RSTG, Right Superior Temporal Gyrus; RLING, Right Lingual Gyrus; RFFG, Right Fusiform Gyrus; GM, Gray Matter; GRF, Gaussian Random Field.

Using the altered rCBF regions as ROIs, the study then performed a seed-based FC analysis (Figures 4, 5). The results showed significant differences in the FC of RMFG with bilateral SOG, right supramarginal gyrus (SMG), and right fusiform gyrus (RFFG) among the groups (Figure 4A). Notably, the bilateral SOG and right SMG showed decreased FC strength in MCI compared to SCD, while the maximum reduction was only observed in the AD group across all regions (Figure 4B). After adjusting the values of *p*, the partial correlation analysis also revealed that the FC strength of RMFG-RSOG significantly affected the visuospatial function in the disease groups (R^2^ = 0.109, *p* = 0.009) (adjusted *p*-values are < 0.01) as shown in Figure 4C.

Moreover, group changes were widely observed between LPCUN and the left median cingulate, between paracingulate gyrus (DCG) and olfactory cortex (OLF), right cuneus (RCUN), superior temporal gyrus (STG), lingual gyrus (LING), and FFG (Figure 5A). Interestingly, although FC was significantly decreased in ADS compared to the HC group, patients with AD displayed an obvious increase in all the different regions compared to the SCD and MCI groups (Figure 5B). Furthermore, the partial correlation analysis revealed significant negative correlations between the FC strength of LPCUN-RCUN and MMSE (R^2^ = 0.177, *p* = 0.001), episodic memory (R^2^ = 0.121, *p* = 0.007), and executive function (R^2^ = 0.131, *p* = 0.005) (adjusted *p*-values are < 0.01) as shown in Figure 5C.

### Analysis of the Altered rCBF, ALFF, and ReHo as Biomarkers in PCC/PCUN

**Figure 6:**
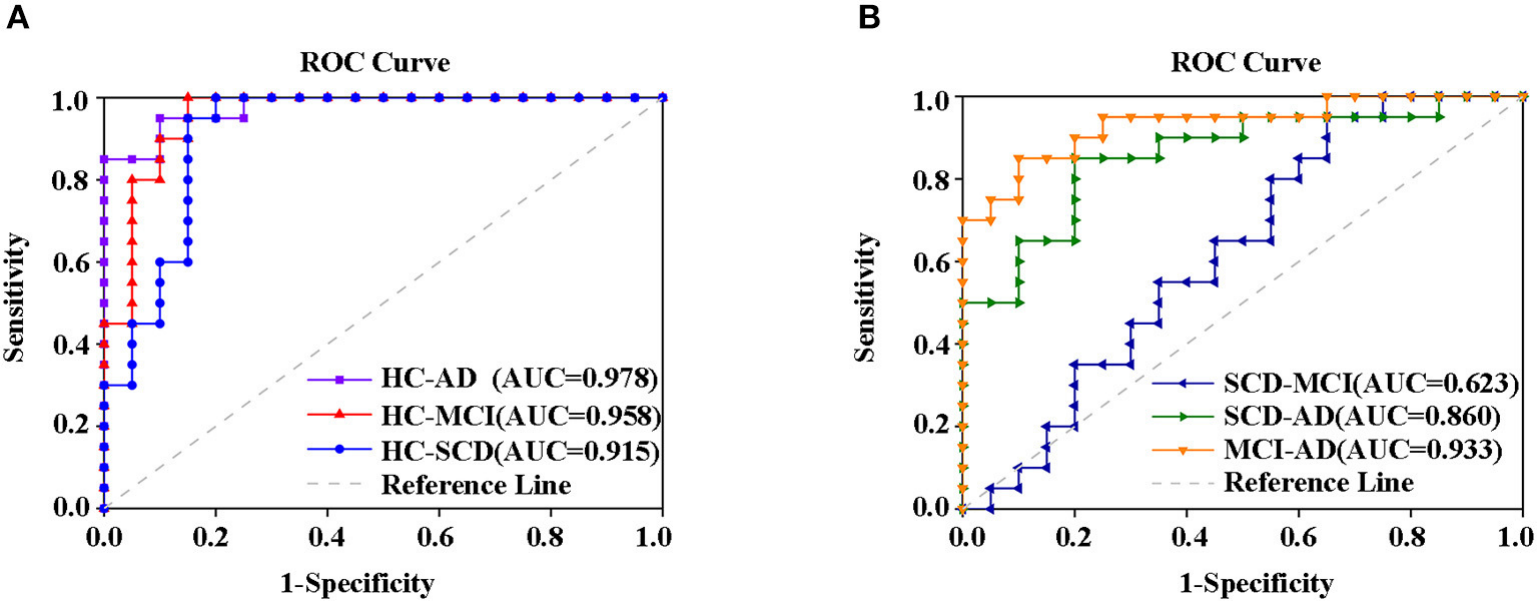
ROC curve from the support vector machine classifier and the general ROC model for classification of the AD spectrum. The results revealed that the combination of altered rCBF, ALFF, and ReHo in PCC/PCUN had more power in differentiating subjects with ADS from HC **(A)** MCI and SCD from patients with AD but not **(B)** SCD from MCI. The classification was able to differentiate disease groups from HC. Specifically, the AUC for the AD patients was 0.978 (95% confidence intervals from 0.942 to 1.000, *p* < 0.001), the MCI had 0.958 (95% confidence intervals from 0.897 to 1, *p* < 0.001), and SCD had 0.915 (95% confidence intervals from 0.82 to 1, *p* < 0.001) **(A)**. Within disease groups, the combination showed a good ability to distinguish AD from MCI (AUC value = 0.933, 95% confidence intervals from 0.855 to 1, *p* < 0.001) as well as AD from SCD (AUC value = 0.86, 95% confidence intervals from 0.744 to 0.977, *p* < 0.001), but not MCI from SCD (AUC value = 0.623, 95% confidence intervals from 0.445 to 0.8, *p* = 0.224) **(B)**. The blue line represents the HC and AD group, the red line represents the HC and MCI group, the purple line represents the HC and AD group, the orange line represents the AD and MCI group, the green line represents the AD and SCD group, the dark blue line represents the MCI and SCD group, and the gray line represents the reference. AD, Alzheimer's Disease; MCI, Mild Cognitive Impairment; SCD, Subjective Cognitive Decline; HC, Healthy Normal; ROC, Receiver Operating Characteristic, AUC, Area Under the Curve; ALFF, Amplitude of Low-Frequency Fluctuation; ReHo, Regional Homogeneity.

Finally, the altered rCBF, ALFF, and ReHo in PCC/PCUN were used to conduct a ROC analysis. The results in Supplementary Figure 2 show that the study was able to differentiate all the disease groups from HC through the classification of altered ReHo (Supplementary Figure 2B) or ALFF (Supplementary Figure 2C) but not altered rCBF, which could only differentiate AD from HC (Supplementary Figure 2A). Within the disease groups, the altered rCBF (Supplementary Figure 2D) and ReHo (Supplementary Figure 2E) were satisfactory in their ability to classify AD and MCI, AD, and SCD but not MCI and SCD. However, the altered ALFF showed the worst performance with regard to classification (Supplementary Figure 2F). Nonetheless, a combination of altered rCBF, ALFF, and ReHo in PCC/PCUN showed a better differentiating ability across the AD spectrum (Figure 6). Therefore, using this classification, the AUC was 0.978 (95% confidence intervals from 0.942 to 1.000, *p* < 0.001), 0.958 (95% confidence intervals from 0.897 to 1, *p* < 0.001), and 0.915 (95% confidence intervals from 0.82 to 1, *p* < 0.001) in the distinction of AD, MCI, and SCD from HC, respectively (Figure 6A). Moreover, the AUC of the difference between MCI and AD was 0.933 (95% confidence intervals from 0.855 to 1, *p* < 0.001) while that of SCD and AD was 0.86 (95% confidence intervals from 0.744 to 0.977, *p* < 0.001). However, the combination displayed a poor ability to differentiate MCI from SCD (AUC value = 0.623, 95% confidence intervals from 0.445 to 0.8, *p* = 0.224) as shown in Figure 6B.

## Discussion

This study aimed to explore the changing patterns in rCBF, brain function, and the behavioral significance across the ADS. First, the results showed that the rCBF in LPCUN and RMFG of the AD group decreased significantly compared to the other three groups. It also had a significant positive association with most cognitive tests except for visuospatial function. Second, the results revealed that the aberrant activity and function were mainly in the posterior brain regions. Compared to the HC group, the ALFF in LPCC/PCUN and the LPCL as well as ReHo in RPCC and RSOG showed an obvious decrease across the disease groups. In addition, ReHo in LMTG, LPCC, and LIPL of the MCI group was higher than that of the AD group. Furthermore, the partial correlation analysis revealed that there was a negative association between ALFF in the LPCL and the MMSE scores, while ReHo in LPCC and LMTG had a positive association with most of the behavioral tests. Additionally, it was shown that the identified regions of the brain had a significant dysfunction in FC and were closely related to cognitive performance. Finally, a combination of altered rCBF, ALFF, and ReHo in PCC/PCUN showed a better differentiating ability across the ADS.

### Differences in rCBF in the ADS

In this study, there was a significant decrease in rCBF in the LPCUN and the RMFC of the AD group. In addition, rCBF was positively associated with cognitive function, consistent with the previous studies (Johnson et al., 2005; Alexopoulos et al., 2012; Hays et al., 2016; Kawagoe et al., 2019; Thomas et al., 2019; Duan et al., 2020). Additionally, a community-based cohort study showed that higher levels of rCBF were associated with better attention, executive function, and memory (Leeuwis et al., 2018). A previous study also showed that the lower the level of rCBF, the worse the cognitive performance was in patients with AD (Leeuwis et al., 2017). Notably, the DMN has two core regions, namely the MFG and the PCUN. The MFG is mainly related to attention, working memory, and regulation of emotions (Seminowicz and Moayedi, 2017), while PCUN is primarily involved in the retrieval of episodic memory, self-consciousness, and processing of the self-relevant effect (Zhang and Li, 2012). Moreover, necropsy revealed that MFG and PCUN were susceptible to Aβ deposition and hypoperfusion in the early stages of AD (Thomas et al., 2015; Miners et al., 2016). According to a previous report, a decrease in CBF starts from the PCUN and propagates along the PCC to other regions of the brain. More importantly, hypoperfusion in these regions showed no significant association with the distribution of brain atrophy in the early onset of familial AD (Benzinger et al., 2013). However, similar hypoperfusion was identified in the late-onset sporadic AD and showed the most pronounced decrease of CBF in PCUN and PCC, as well as in the prefrontal, parietal, and occipital cortices (Binnewijzend et al., 2013). Moreover, CBF in the prefrontal cortex was shown to be highly sensitive in the prediction of future cognitive performance (De Vis et al., 2018), while decreased CBF in the PCUN was considered to be a marker of severity in cognitive impairment (Binnewijzend et al., 2013). Intriguingly, aberrant local perfusion in the brain revealed that neurovascular dysfunction is commonly present in ADS. This also implied that the reduction in rCBF may be closely related to the progression of pathological processes in AD (Leeuwis et al., 2017), forming a vicious circle. Notably, decreased brain perfusion reduces the clearance of Aβ, leading to the accumulation of amyloid plaques and neurofibrillary tangles, which further impair vascular function and exacerbate the reduction of CBF (Popa-Wagner et al., 2015). As such, the altered rCBF may interfere with brain function and aggravate cognitive decline in the ADS.

### Changes in Brain Activity and FC Among the Subjects

The study identified multiple areas of the brain with decreased activity and disrupted FC (Liu et al., 2008; Han et al., 2012; Zhang et al., 2012; Pan et al., 2017; Min et al., 2019). Additionally, two recent meta-analyses demonstrated that the decreased ALFF and ReHo in patients with MCI were primarily located in the BPCC/PCUN, bilateral frontal, left occipitotemporal cortex, and parietal lobule compared to HC (Pan et al., 2017; Zhen et al., 2018). In the present study, the findings showed that the altered regions were mainly located in the posterior areas of the brain, including the PCC/PCUN, LMTG, IPL, SOG, FFG, and LING. These constitute parts of the DMN, the executive control network (ECN), and the visual network (VN) (Pan et al., 2017; Zhen et al., 2018). Moreover, numerous studies reported on the interruption of the connectivity of DMN, ECN, and VN in MCI/AD (Bokde et al., 2006; Sorg et al., 2007; Brier et al., 2012; Wang et al., 2015; Joo et al., 2016; Eyler et al., 2019). It is also well-known that visual impairment is one of the most important clinical signs of AD and accounts for about 30% in MCI (Mapstone et al., 2003) and up to 50% in AD (Mendola et al., 1995). Additionally, a marked decrease in glucose metabolism was reported in the parietal and occipital cortices of patients with AD (Pietrini et al., 1996). Existing evidence suggests that visual impairment might arise from the abnormal connectivity of the VN and other regions of the brain (Bokde et al., 2006; Vannini et al., 2008; Zheng et al., 2019). However, it is important to note that both brain activity and FC significantly decreased as early as in SCD. This suggests that dysregulation of brain neuronal excitability appears before objective impairment upon formal testing and that might be a potential biomarker (Mattson and Arumugam, 2018; Si et al., 2020). Interestingly, the FC strength between LPCUN and the altered regions of the brain in the AD group showed an increasing trend compared to the SCD and MCI groups. Moreover, there was a significant negative correlation between the FC of LPCUN-RCUN and MMSE, episodic memory, and executive functioning. This may have been due to the slight global cognitive impairment in the preclinical stage of AD, which only manifested as decreased FC. However, the FC in the core hub of DMN led to a compensatory rise in order to maintain cognitive function during the progression of the disease (Qi et al., 2010). It is noteworthy that with continued amyloid deposition and substantial loss of neurons in the late stages of AD, DMN gradually falls out of the compensatory mode, leading to a severe decrease in FC (Tuovinen et al., 2016; Scherr et al., 2019). Furthermore, the results showed that the ALFF in LPCL had a negative association with the MMSE score. The PCL is located in the posterior ventral region of the inferior frontal gyrus (IFG), which is a crucial cortical node for the cognitive control in the circuits. Additionally, with the aggravation of cognitive impairment, the cortical motor regions in the circuits compensate for the damaged brain function by part activation (Zhang et al., 2020). In summary, the results suggested that there is a significant difference in brain activity and FC across the ADS, and the difference appears as early as in SCD. Moreover, the difference is closely correlated with cognitive performance and can be used as a potential imaging biomarker for monitoring disease progression in AD.

### Altered rCBF, ALFF, and ReHo as Biomarkers in PCC/PCUN

Numerous studies have reported on the altered rCBF, the deposition of AD pathology biomarkers, and decreased brain function in the PCC/PCUN of AD (Benzinger et al., 2013; Aghakhanyan et al., 2018; Zhu et al., 2019). In addition, the rCBF was validated in a previous study as a diagnostic marker for AD but not for preclinical AD (Zheng et al., 2019). It is well-known that the machine learning method is widely employed for classification in clinical research and has been used for the prediction of AD and preclinical AD (Liu et al., 2013; Xu et al., 2018). Therefore, using SVM, the study performed an integrated analysis of altered rCBF, ALFF, and ReHo in PCC/PCUN as biomarkers to uncover the differentiating power across the AD spectrum. The results revealed that a combination of the three measured brain functional changes in PCC/PCUN has a better differentiating power across the ADS compared to each parameter. Notably, the combination was able to differentiate the disease groups from HC. Moreover, the combination displayed a good ability to distinguish between AD and MCI, AD and SCD, but not MCI and SCD in the disease groups. Therefore, the study demonstrated that a combination of multimodal neuroimaging in PCC/PCUN might be an effective biomarker for differentiating the ADS.

## Limitations

Although the study supplemented and refined similar reports from the past, it had a number of limitations. First, there was no evidence of a biomarker for amyloid pathology and genetic data. Secondly, the study was a single-center cross-sectional study and might therefore have presented insufficient data. Thirdly, the sample size was relatively small and some analyses may have had insufficient power. Moving forward, more volunteers will be recruited to participate in the study. In addition, the study will refine the detection of pathological markers of AD and obtain genetic data. Finally, regular follow-up will be conducted in the future in order to further confirm the results.

## Conclusion

This study demonstrated obvious changes in CBF, brain activity, and FC in the ADS, and these could appear in the early stages of the disease. In addition, a disrupted FC, decreased CBF, and brain activity were associated with more serious cognitive impairment, reflecting brain neurovascular dysfunction in ADS. Finally, the combination of altered rCBF, ALFF, and ReHo in PCC/PCUN proved to be a powerful tool in differentiating the ADS and is therefore a potential neuroimaging biomarker.

## Data Availability Statement

The raw and processed data in this study cannot be shared at this time due to the ethics and the protection of privacy issues of the participants. And these data also forms part of an ongoing study.

## Ethics Statement

The studies involving human participants were reviewed and approved by Research Ethics Committee of the Affiliated ZhongDa Hospital, Southeast University (Nanjing, China). The patients/participants provided their written informed consent to participate in this study.

## Author Contributions

ZZ, ZW, and CX were responsible for coordinating the entire data collection process and checking the quality of data during the collection period. YZ, FZ, CT, SZ, and HS were responsible for data collection. QW, CH, and DF were responsible for cognitive function assessment and neurological examination. QZ made a draft of the manuscript. HF polished the manuscript and language revision. All authors read and approved the final manuscript.

## Conflict of Interest

The authors declare that the research was conducted in the absence of any commercial or financial relationships that could be construed as a potential conflict of interest.
