# Supplementary material for: Altered Regional Cerebral Blood Flow and Brain Function Across the Alzheimer's Disease Spectrum: A Potential Biomarker
Source: Front Aging Neurosci. 2021 Feb 22;13:630382. doi: 10.3389/fnagi.2021.630382 (PMC7937726; doi:10.3389/fnagi.2021.630382)
Supplement: Supplementary file 1 [file Table_1.DOCX]

Supplementary Material

# SUPPLEMENTARY METHODS

***Statistical analysis***

***Classification with the Support Vector Machine***

The classification was conducted using linear support vector machine (SVM) to differentiate subjects with ADS from HC, MCI from SCD, AD from MCI and AD from SCD. In the implementation, we used a SVM package whcih was bult in the MATLAB, LIBSVM toolbox (Chang and Lin, 2011), to get optimal classifiers and test the power of classification. SVM classification is a form of supervised learning because training is accomplished by labeled samples (Burges, 1998). A binary label with -1 for patients (AD, MCI and SCD) and 1 for HC subjects was used in this study. Two steps were conducted in the classification process: training and testing. During the training step, the SVM calculates a decision boundary that separates the samples in the input space using their class labels. Once the decision function is determined from the training set, it can be used to predict the class label of a new testing example.

Evidence suggests that SVM with linear kernel is an appropriate model to avoid over-fitting and to allow direct extraction of the feature weights when training samples are small but the features number are large (Patel et al., 2016; Pereira et al., 2009; Vapnik, 1995). The linear SVM has only one parameter C, which determines the trade-off between the minimization of training error and misclassification penalty, was set to the default value (C = 1) for all cases.

Finally, due to our limited number of samples, leave-one-out cross-validation (LOOCV) was used to quantify the classification performance (Wee et al., 2011). The performance of classifier was also assessed using receiver operating characteristics (ROC) curves from the results of the LOOCV data. The area under the ROC curve (AUC) represents the classification power of a classifier, with a larger AUC indicating a better classification power.

References:

Burges, C., 1998. A tutorial on support vector machines for pattern recognition. Data Min Knowl Disc, 121-167.

Chang, C., Lin, C., 2011. LIBSVM: a library for support vector machines. ACM Trans Intell Syst Technol (TIST), 2-27

Patel, M.J., Khalaf, A., Aizenstein, H.J., 2016. Studying depression using imaging and machine learning methods. Neuroimage Clin 10, 115-123.

Pereira, F., Mitchell, T., Botvinick, M., 2009. Machine learning classifiers and fMRI: a tutorial overview. Neuroimage 45, S199-209.

Vapnik, V., 1995. The nature of statistical learning theory. Springer, New York Inc;.

Wee, C.Y., Yap, P.T., Li, W., Denny, K., Browndyke, J.N., Potter, G.G., Welsh-Bohmer, K.A., Wang, L., Shen, D., 2011. Enriched white matter connectivity networks for accurate identification of MCI patients. Neuroimage 54, 1812-1822.

# Supplementary Figures and Tables

**Supplementary Table 1.** **Neuropsychological features of all subjects.**

|  | HC(n = 20) | SCD(n = 20) | MCI(n = 20) | AD(n = 20) | *p* Value |  |
| --- | --- | --- | --- | --- | --- | --- |
| AVLT-20-min-DR | 1.05±0.53 | 0.69±0.63 | -0.84±0.26^bd^ | -0.91±0.34^ce^ | <0.001 |  |
| LMT-20-min-DR | 0.89±0.68 | 0.42±0.92 | -0.28±0.53^bd^ | -1.11±0.37^cef^ | <0.001 |  |
| ROCFT-20-min-DR | 0.81±0.71 | 0.26±0.88 | -0.40±0.84^bd^ | -0.84±0.51^ce^ | <0.001 |  |
| CDT | 0.28±0.61 | 0.18±0.97 | 0.30±0.46 | -0.78±1.35^cef^ | 0.001 |  |
| ROCFT-IR | 0.37±0.20 | 0.31±0.44 | -0.17±1.05 | -0.41±1.47 | 0.027 |  |
| DSST | 0.67±0.67 | 0.34±0.79 | 0.03 ±0.80 | -0.97±0.79^cef^ | <0.001 |  |
| TMTA | 0.50±0.69 | 0.37±0.77 | 0.11±0.96 | -1.02±0.87^cef^ | <0.001 |  |
| Stroop Color and Word Test A | 0.60±0.79 | 0.30±0.79 | 0.05±0.90 | -1.06±0.88^cef^ | <0.001 |  |
| Stroop Color and Word Test B | 0.69±0.83 | 0.16±0.84 | 0.12±0.85 | -1.05±0.80^cef^ | <0.001 |  |
| VFT | 1.09±0.68 | 0.02±0.40^a^ | 0.07±0.70^b^ | -1.13±0.59^cef^ | <0.001 |  |
| DST | 0.20±0.92 | 0.24±0.71 | 0.32 ±0.87 | -0.63±0.97^f^ | 0.002 |  |
| TMT-B | 0.31±0.71 | 0.53±0.85 | 0.01±0.80 | -1.07±0.61^cef^ | <0.001 |  |
| Stroop Color and Word Test C | 0.20±0.35 | 0.05±0.43 | 0.01±0.33 | -0.73±0.41^cef^ | <0.001 |  |
| Semantic Similarity test | 0.69±0.60 | 0.01±0.78 | 0.22 ±0.57 | -0.79±1.14^cef^ | <0.001 |  |

**Note:** Data was presented as the mean ± standard deviation (SD). p values were obtained by one-way ANOVA (age and education)and ANCOVA (controlled age, gender, and education) analysis. a-f) post-hoc analysis (Bonferroni correction) further revealed the source of ANCOVA difference. a) HC versus SCD; b) HC versus MCI; c) HC versus AD; d) SCD versus MCI; e) SCD versus AD; f) MCI versus AD).

**Abbreviations:** HC: healthy control, SCD: subjective cognitive decline, MCI: mild cognitive impairment, AD: Alzheimer’s disease, F/M: female/male, MMSE: Mini-Mental State Examination, ADL: Activities of daily living, HAMD: Hamilton Depression Scale, HIS: Hachinski Ischemic Scale.AVLT- 20-min-DR: Auditory Verbal Learning Test-20-minutes Delayed Recall, LMT-20-min-DR: Logical Memory Test-20-minutes Delayed Recall, ROCFT-20-min-DR: Rey-Osterrieth Complex Figure Test-20- minutes Delayed Recall, DSST: Symbol Digit Modalities Test, TMT-A: Trail Making Tests-A, CDT: Clock Drawing Test, ROCFT: Rey-Osterrieth Complex Figure Test, DST: Digit Span Test, VFT: Verbal Fluency Test, TMT-B: Trail Making Tests B.

**Supplementary Table 2. Brain regions with altered CBF, ALFF, Reho, and FC in all subjects.**

| Location(AAl) | MNI coordinate | | | Voxel | Cluster size | Statistical value(F) |
| --- | --- | --- | --- | --- | --- | --- |
|  | X | Y | Z |  | (mm3) |  |
| **Brain regions with different CBF** | | | | | | |
| RMFG | 32 | 4 | 56 | 26 | 208 | 7.77 |
| LPCUN | -8 | -70 | 42 | 129 | 1032 | 10.76 |
| **Brain regions with different ALFF** | | | | |  | |
| LPCC | -6 | -39 | 9 | 10 | 270 | 10.1 |
| LPCL | -12 | -27 | 63 | 10 | 270 | 11.42 |
| LPCUN | -6 | -42 | 66 | 23 | 621 | 9.02 |
| **Brain regions with different Reho** | | | | |  | |
| RSOG | 21 | -72 | 45 | 37 | 999 | 11.9 |
| RPCC | 18 | -51 | 15 | 22 | 594 | 10.83 |
| LPCC | -6 | -51 | 36 | 18 | 486 | 8.34 |
| LIPL | -27 | -51 | 42 | 16 | 432 | 11.67 |
| LMTG | -51 | -54 | 24 | 10 | 270 | 8.46 |
| **Brain regions with different FC** | | | | |  | |
| RMFG-RSMG | 45 | -33 | 36 | 10 | 270 | 7.74 |
| RMFG-RFFG | 39 | -57 | -9 | 14 | 378 | 9.04 |
| RMFG-RSOG | 24 | -60 | 33 | 21 | 567 | 7.84 |
| RMFG-LSOG | -21 | -63 | 30 | 13 | 351 | 9.81 |
| LPCUN-RSTG | 48 | -30 | 15 | 43 | 378 | 10.08 |
| LPCUN-RLING | 57 | -12 | -21 | 7 | 1161 | 8.09 |
| LPCUN-RFFG | 27 | -60 | -15 | 17 | 189 | 8.35 |
| LPCUN-RCUN | 9 | -84 | 21 | 35 | 459 | 9.09 |
| LPCUN-LDCG | -18 | -36 | 45 | 20 | 945 | 10.34 |
| LPCUN-LOLF | -15 | 15 | -18 | 16 | 540 | 11.38 |


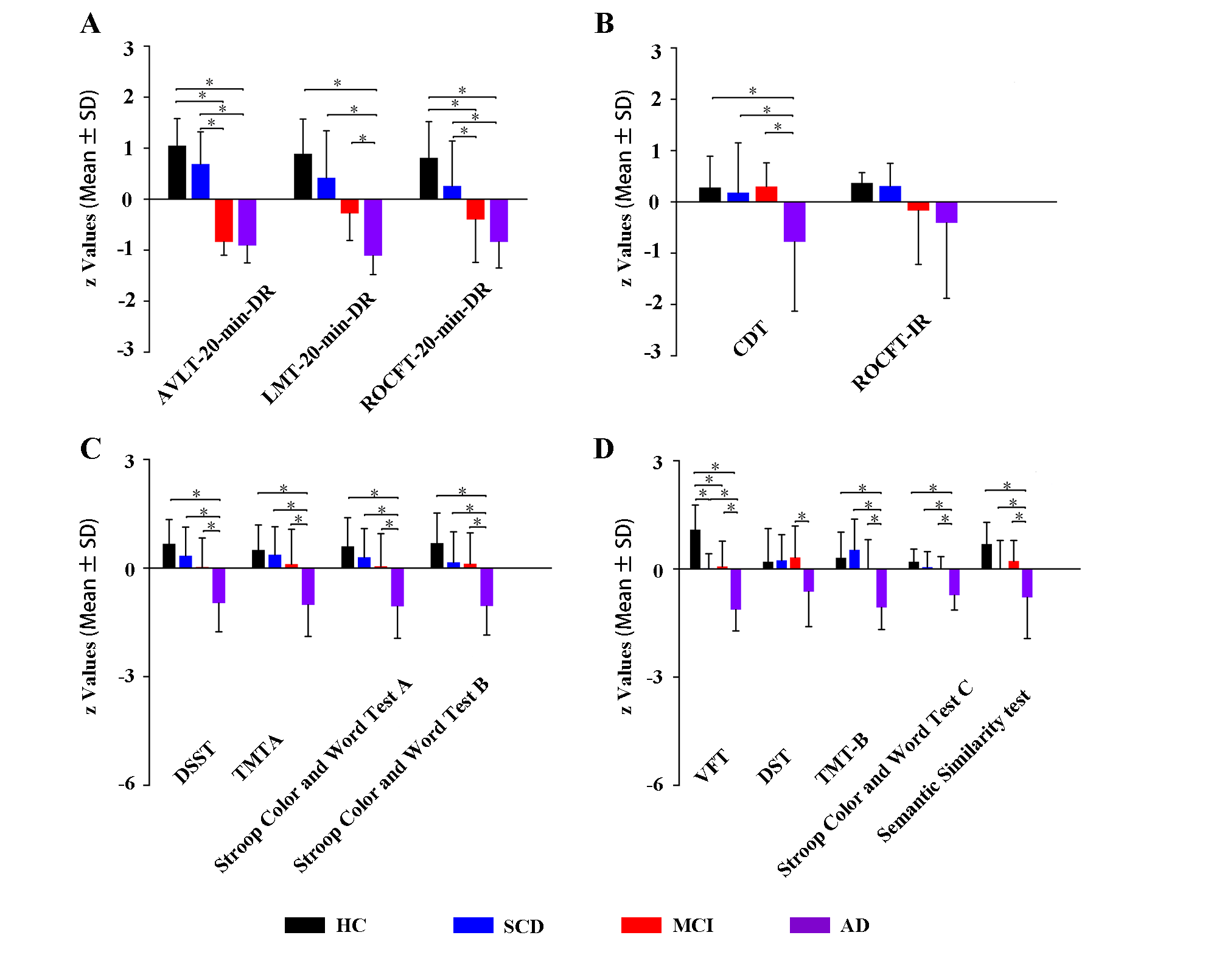


**Supplementary Figure1**. Group-level differences and Post-hoc analysis in neuropsychological features of all subjects. A) Episodic memory, including AVLT-20-min-DR, LMT-20-min-DR, and ROCFT-20-min-DR; B) Visuospatial function, including CDT, ROCFT-IR; C) Information processing speed, including DSST, TMTA, Stroop Color and Word Test A, Stroop Color and Word Test B; D) Executive function, including VFT, DST, TMT-B, Stroop Color and Word Test C, Semantic Similarity test. Bonferroni correction, p<0.05. Error bars represent the standard deviation. * represents statistical difference (p<0.05).

**Abbreviations:** HC: healthy control, SCD: subjective cognitive decline, MCI: mild cognitive impairment, AD: Alzheimer’s disease, F/M: female/male, MMSE: Mini-Mental State Examination, ADL: Activities of daily living, HAMD: Hamilton Depression Scale, HIS: Hachinski Ischemic Scale.AVLT- 20-min-DR: Auditory Verbal Learning Test-20-minutes Delayed Recall, LMT-20-min-DR: Logical Memory Test-20-minutes Delayed Recall, ROCFT-20-min-DR: Rey-Osterrieth Complex Figure Test-20- minutes Delayed Recall, DSST: Symbol Digit Modalities Test, TMT-A: Trail Making Tests-A, CDT: Clock Drawing Test, ROCFT: Rey-Osterrieth Complex Figure Test, DST: Digit Span Test, VFT: Verbal Fluency Test, TMT-B: Trail Making Tests B.


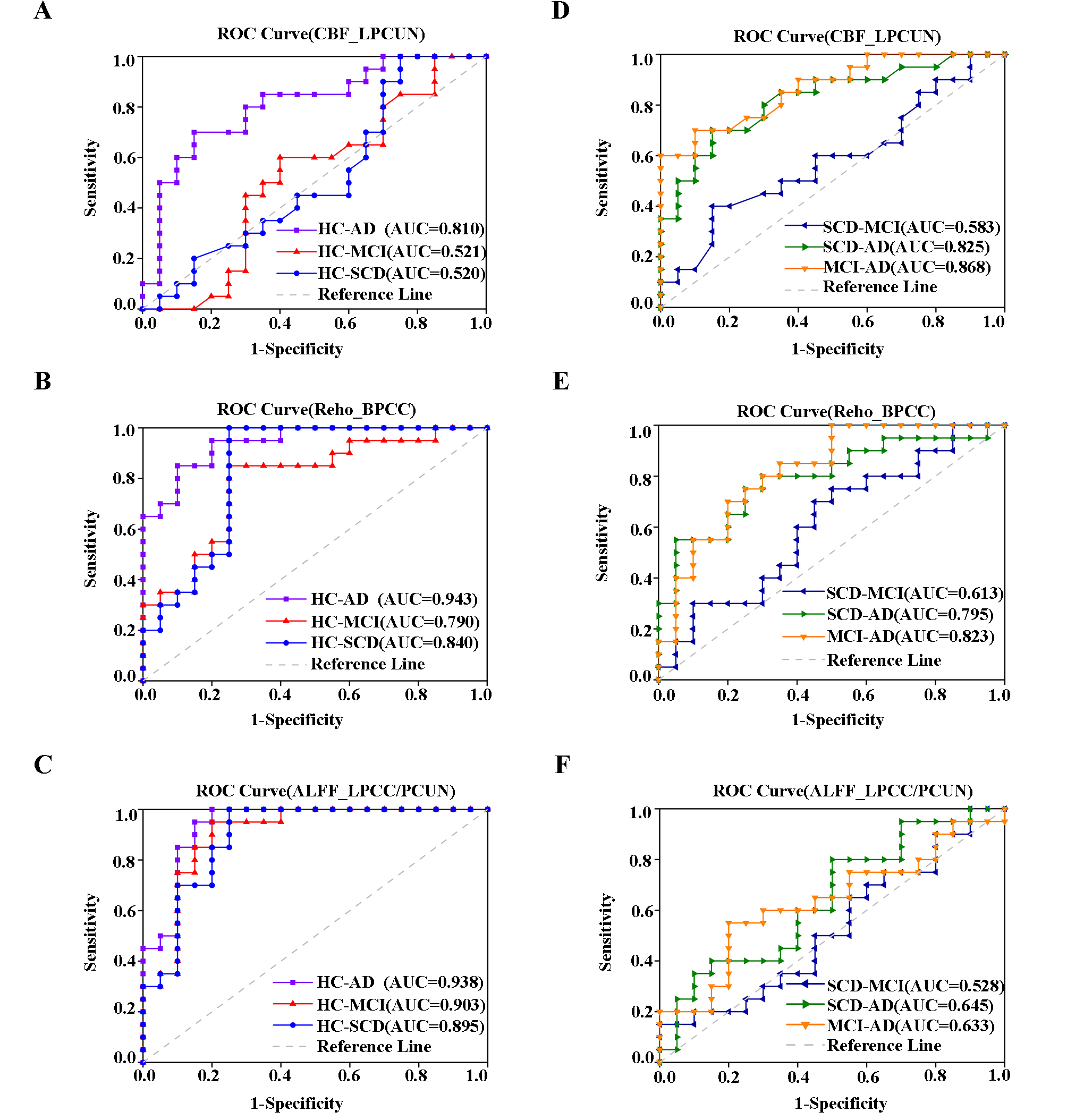


**Supplementary** Figure2**. ROC curve for classifying AD spectrum patients using altered rCBF, ALFF, and Reho respectively.** We were able to differentiate all disease groups from HC with the classifation of altered Reho(B) or ALFF(C), but not with alterd rCBF, which could only differentiate AD from HC(A). Within disease groups, the altered rCBF(D) and Reho(E) showed good power for classifying AD and MCI, AD and SCD, but not for MCI and SCD, while with the altered ALFF, it showed the poorest classification performance(F). Concretely, between HC and disease groups, A) CBF_LPCUN : the AUC for the AD patients was 0.810 (95% confidence intervals from 0.674 to 0.946, p<0.001), for the MCI was 0.521 (95% confidence intervals from 0.335 to 0.707, p=0.818), and for the SCD was 0.520 (95% confidence intervals from 0.335 to 705, p=0.829). B) Reho_BPCC: the AUC for the AD patients was 0.942 (95% confidence intervals from 0.877 to 1, p<0.001), for the MCI was 0.790 (95% confidence intervals from 0.648 to 0.932, p=0.002), and for the SCD was 0.840 (95% confidence intervals from 0.707 to 0.973, p<0.001). C) ALFF_LPCC/PCUN : the AUC for the AD patients was 0.938 (95% confidence intervals from 0.861 to 1, p<0.001), for the MCI was 0.902 (95% confidence intervals from 0.803 to 1, p<0.001), and for the SCD was 0.895 (95% confidence intervals from 0.792 to 0.998, p<0.001). Within disease groups, D) CBF_LPCUN :AD and MCI (AUC value =0.868, 95% confidence intervals from 0.759 to 0.976, p<0.001), AD and SCD (AUC value=0.825, 95% confidence intervals from 0.696 to 0.954, p<0.001), MCI and SCD (AUC value=0.583, 95% confidence intervals from 0.403 to 0.762, p=0.372) . E) Reho BPCC: AD and MCI (AUC value=0.822, 95% confidence intervals from 0.693 to 0.952, p<0.001), AD and SCD (AUC value=0.795, 95% confidence intervals from 0.654 to 0.936, p=0.001), MCI and SCD (AUC value=0.612, 95% confidence intervals from 0.436 to 0.789, p=0.224). F) ALFF_LPCC/PCUN: AD and MCI (AUC value=0.633, 95% confidence intervals from 0.456 to 0.809, p=0.152), AD and SCD (AUC value=0.645, 95% confidence intervals from 0.473 to 0.817, p=0.117), MCI and SCD (AUC value=0.528, 95% confidence intervals from 0.345 to 0.710, p=0.766). Blue line represents HC and AD group, red line represents HC and MCI group, purple line represents HC and AD group, orange line represents AD and MCI group, green line represents AD and SCD group, dark blue line represents MCI and SCD group, and gray line represents the reference line. Abbreviations: AD: Alzheimer's disease; MCI: mild cognitive impairment; SCD: subjective cognitive decline; HC: health normal; CBF: cerebral blood flow; ReHo: regional homogeneity; ALFF: amplitude of low frequency fluctuation; LPCUN: left precuneus; BPCC: bilateral posterior cingulate cortex; ROC: receiver operating characteristic; AUC: area under the curve.
